# Sex differences in the relationship of biomarker change to memory decline in early Alzheimer’s disease: an observational cohort study

**DOI:** 10.1186/s13293-025-00820-6

**Published:** 2026-01-16

**Authors:** Erin E. Sundermann, Sarah J. Banks, Mark W. Bondi, Maricedes Acosta Martinez, Anat Biegon, Lindsay J. Rotblatt, Thomas Hildebrandt

**Affiliations:** 1 Department of Psychiatry, University of California, 9500 Gilman Drive, San Diego, La JollaCA Mail Code 8218 USA; 2 Department of Neurosciences, University of California, 9500 Gilman Drive, San Diego, La JollaCA Mail Code 0662 USA; 3 San Diego Healthcare System, San Diego, CA USA; 4 Department of Physiology and Biophysics, Stonybrook University, 101 Nicolls Road, BST 5-180, Stony Brook, NY USA; 5 Department of Women’s health, Uppsala University, Akademiska sjukhuset 751 85, Uppsala, Sweden; 6 Department of Psychiatry, Mt. Sinai School of Medicine, 1 Gustave L. Levy Place, Box 1230 NY, USA; 7 UCSD Altman Clinical and Translational Research Institute, office 2W517, 9452 Medical Center Dr. (MC 0875), La Jolla, CA USA

**Keywords:** Sex/gender, Alzheimer’s disease, Mild cognitive impairment, Preclinical alzheimer’s disease, Alzheimer’s pathology, Verbal memory, Clinical presentation, Cognitive reserve

## Abstract

**Background:**

Alzheimer’s disease (AD) exhibits sex differences in pathology and cognitive trajectories. Understanding how these differences manifest across the Alzheimer’s continuum can improve early detection, diagnostics, and interventions. We examined sex differences in the association between cerebrospinal fluid (CSF) pTau181/Aβ42 ratio changes and verbal memory decline across the preclinical and mild cognitive impairment (MCI) stages of AD.

**Methods:**

In this retrospective, longitudinal, observational study, data were extracted from 401 participants (age range: 55-87.8, 98% non-Hispanic White) of the Alzheimer’s Disease Neuroimaging Initiative cohort study who were classified as either preclinical AD (78 females, 73 males) or MCI (104 females, 146 males) at baseline and had CSF pTau181/Aβ42 ratio and cognitive assessment data at at-least two timepoints. Using regression models, we examined the relationship between changes in CSF pTau181/Aβ42 and verbal memory across all available time points and the moderating role of sex and AD stage over a mean follow-up period of 4 years. Verbal memory was represented by a composite z-score averaging Learning and Delayed Recall z-scores of the Rey Auditory Verbal Learning Test. Covariates included baseline age, education, and apolipoprotein E genotype.

**Results:**

A significant sex * diagnostic group * biomarker change interaction (*b*=-17.47, 95%CI = 27.60 to -7.33, *p* = .001) indicated that sex differences in the relationship between changes in CSF pTau181/Aβ42 ratio and verbal memory differed by disease stage. While males in the preclinical AD stage showed steeper memory decline than females with increasing pTau181/Aβ42 ratios, this difference was not statistically significant. In contrast, in the MCI stage, a significant sex * biomarker change interaction (*b* = 10.17, 95% CI = 4.94 to 15.40, *p* < .001) indicated that females exhibited significantly steeper memory decline associated with increasing pTau181/Aβ42 ratios compared to males.

**Conclusion:**

Sex differences in the relationship between AD biomarker levels and cognitive decline vary by disease stage. Although not statistically significant, females demonstrated resilience to memory decline in the preclinical stage, whereas, in the MCI stage, they experienced significantly steeper memory loss compared to males. Results suggest that accounting for sex in biomarker-based methods of disease detection and tracking can improve early detection and intervention in both sexes.

**Supplementary Information:**

The online version contains supplementary material available at 10.1186/s13293-025-00820-6.

## Highlights

Alzheimer’s disease (AD) biomarkers in cerebrospinal fluid (pTau181/Aβ42) were linked to verbal memory decline differently in women and men, showing that sex modifies biomarker–cognition relationships across disease stages.

Despite women showing more resilience to memory decline than men as the AD biomarker advanced in the preclinical stage, this pattern significantly reversed at the MCI stage, whereby.

women showed significantly faster memory decline than men as the AD biomarker advanced.

Findings suggest a possible “tipping point” in women, where early resilience transitions to greater vulnerability as AD progresses.

Results underscore the importance of considering sex when using biomarkers to detect, track, and treat AD.

**Supplementary Information:**

The online version contains supplementary material available at 10.1186/s13293-025-00820-6.

## Abstract (plain language summary)

Alzheimer’s disease (AD) affects men and women differently, but the reasons why remain unclear. Women are more likely to develop AD and often show higher levels of tau, a protein linked to brain changes in the disease. At the same time, women tend to have better memory than men in the very early stages, which may delay diagnosis until the disease has progressed further.

In this study, we analyzed data from 401 older adults in the Alzheimer’s Disease Neuroimaging Initiative. Participants were either in the “preclinical” stage of AD (biomarker changes but no memory problems) or had mild cognitive impairment (MCI). We measured changes in spinal fluid proteins (pTau181/Aβ42 ratio) linked to AD and tracked how they related to changes in memory performance over about four years.

We found that sex differences depended on the stage of disease. In the preclinical stage, men tended to show faster memory decline than women as AD biomarkers worsened, although this was not statistically significant. In contrast, among people with MCI, women showed significantly faster memory decline than men as biomarkers increased.

These findings suggest that women may be more resilient to early Alzheimer’s-related brain changes but reach a “tipping point” at which their memory declines faster once symptoms begin. Considering sex when interpreting AD biomarkers may improve early detection, diagnosis, and treatment for both men and women.

**Supplementary Information:**

The online version contains supplementary material available at 10.1186/s13293-025-00820-6.

## Background

The two-fold higher prevalence of Alzheimer’s disease (AD) in females is well-established [1]. Alzheimer’s disease is characterized by the hallmark pathologies of amyloid-beta plaques (Aβ) and phospho-Tau (p-Tau)-based neurofibrillary tangles followed by neurodegeneration. These pathologies form the basis of the A/T/N framework used for diagnosis and staging through biomarkers of Aβ deposition, tau pathology, and neurodegeneration. Many studies have examined sex differences in the burden of AD pathology and generally find that females typically show a greater burden of pathological tau than males, particularly among amyloid positive individuals who are either cognitively normal or have mild cognitive impairment (MCI) [2–6]. However, how the clinical presentation of AD pathology may differ for males and females is a question far less examined, although equally important to understand in terms of improving early detection, intervention and disease tracking in each sex.

Evidence so far suggests that, despite the greater tau burden in females compared to males, females tend to have a cognitive advantage over men in the preclinical AD stages (i.e., cognitively normal but AD biomarker positive) to early MCI stage of the AD continuum, particularly on tests of verbal memory [7–12]. Cross-sectional and longitudinal studies have reported that cognitively normal and amyloid-positive females often maintain better memory performance than males, but this advantage diminishes or reverses as pathology markers progress and clinical symptoms emerge memory [7–15]. This pattern implies that females may initially demonstrate resilience to AD pathology but experience a steeper decline once clinical symptoms manifest.

Longitudinal studies have supported the steeper decline in females at intermediate disease stages as measured by clinical symptom severity or more advanced pathology burden [13–15].

Other studies have consistently reported sex differences in how baseline AD biomarkers relate to cognitive trajectories among older cognitively unimpaired adults [16, 17]. Overall, prior literature suggests sex differences in the relationship between AD pathology and clinical symptoms, with patterns observed across studies implying that these differences shift with disease stage. Yet, much of the existing work has relied on either cross-sectional comparisons across diagnostic stages or longitudinal cognitive outcomes anchored to baseline biomarker levels. These designs cannot fully capture how within-person changes in biomarkers relate to within-person changes in cognition, nor whether these biomarker–cognition linkages differ by sex and/or disease stage.

We address a critical knowledge gap by directly testing sex differences in how longitudinal changes in AD biomarkers track with longitudinal cognitive change and whether these differences vary by disease stage. Such analyses are essential to determine whether the mechanisms linking advancing pathology to cognitive decline operate differently in men and women and whether those differences shift across disease stages. Specifically, we examined sex differences in the relationship of changes in cerebrospinal fluid (CSF) pTau_181_/Aβ_42_ ratio to changes in verbal memory and the moderating role of Preclinical AD versus MCI disease stage among Alzheimer’s Disease Neuroimaging Initiative (ADNI) participants. We chose this marker rather than examining Aβ and tau biomarkers individually because the ratio captures the interplay between Aβ and tau pathologies and this interplay is more closely tied to disease progression and cognitive decline [18–20] and shows critical sex differences [4]. We hypothesized that increases in the pTau_181_/Aβ_42_ ratio are associated with greater memory decline in males relative to females among Preclinical AD individuals but the opposite is true in MCI individuals, in whom increases in the pTau_181_/Aβ_42_ ratio are associated with greater memory decline in females compared to males.

## Methods

### Participants and data source

Data collected between 2005 and 2019 were extracted from the ADNI database. ADNI data is publicly available at adni.loni.usc.edu. ADNI is a longitudinal, multi-site, cohort study that began in 2003 as a public-private partnership and recruited a cohort of healthy older adults (age ≥ 55), and individuals with MCI and early AD type dementia. Information about ADNI can be found at www.adni-info.org. ADNI study visits involve neuroimaging, neuropsychological and clinical assessments. The general enrollment inclusion/exclusion criteria for ADNI have been described elsewhere [21]. This specific study included participants who met the following inclusion criteria: (1) classified as either preclinical AD or MCI at baseline; (2) had CSF AD biomarker and cognitive assessment data at baseline as well as at-least one follow-up visit; (3) had relevant covariate data at baseline (e.g., demographics, apolipoprotein E ε4 [APOE-ε4] status. This resulted in a sample of 401 older individuals (age range: 55–88) classified as preclinical AD (78 females, 73 males) or MCI (104 females, 146 males) at baseline.

### Diagnostic classification

Diagnosis of cognitively normal versus MCI was based on the Jak/Bondi classification method [22]. Applied to ADNI, the Jak/Bondi diagnosis includes six neuropsychological tests representing three cognitive domains: Trail-Making Tests A and B (psychomotor speed/executive function), Category Fluency and Boston Naming Test (language) and the RAVLT Delayed Recall and Recognition Tests (episodic memory). Z-scores were calculated for individual neuropsychological measures using predicted values relative to an ADNI robust normal control group (i.e., diagnosed as cognitively normal at all of the participant’s ADNI visits, *n* = 525) that adjusted for age, sex, and education in a normative regression procedure. An impaired score was defined as > 1 SD below the age-, sex- and education- corrected normative mean. MCI diagnosis was provided if the participant did not meet diagnostic criteria for dementia based on the standard NINCDS/ADRDA criteria [23] and met one of two of the following criteria: (1) impaired score on two tests within a cognitive domain or (2) one impaired score in each of the three cognitive domains. Participants who did not meet one of these two criteria were classified as cognitively normal. Among cognitively normal individuals, those who were positive for either CSF p-Tau/Aβ ratio, amyloid PET or Tau PET biomarkers based on established cut-points [24–26] were classified as preclinical AD.

### Cognitive outcome

ADNI study visits include a battery of neuropsychological tests. We chose episodic memory as our outcome of interest because tests of verbal memory are most often used in MCI/AD diagnostic protocols and provide robust sensitivity to cognitive decline across the AD continuum given their early and robust declines. Episodic memory was measured via a verbal memory test, the Rey Auditory Verbal Learning Test (RAVLT). The RAVLT involves learning a list of 15 unrelated words and immediately recalling as many words as possible across five learning trials (“Learning score”, range: 0–75), after learning an interference list, and after a 30-minute delay period (“Delayed Recall score”, range: 0–15). Within the study sample, the Learning and Delayed Recall scores were standardized (z-scored) and then averaged together to create a verbal memory composite score. The RAVLT has previously shown a strong female advantage [27]. Change score was calculated by creating lagged residuals with a lag of one time point.

### CSF AD biomarker

We examined the ratio of CSF levels of hyperphosphorylated tau-181 (p-tau181) to Aβ1–42 proteins (pTau_181_/Aβ_42_ ratio). To model change in biomarker levels, we generated lagged residuals by predicting each biomarker value from its value at the prior visit (lag of one time point). The residuals from these models—representing deviation from the expected value based on prior levels—were then used as the measure of biomarker change. This approach reduces bias from autocorrelation in repeated measures.

### Statistical analyses

Differences in sample characteristics and variables of interest at baseline between sexes were examined using independent t-tests for continuous variables and Chi-square tests for categorical variables. Regression models were built using lme4 package [28] in R v4.1.3 [29] to test for the effect of change in CSF pTau_181_/Aβ_42_ biomarker levels on the verbal memory composite score. To reduce potential sources of sampling bias, we included age, education, and income in regression models.

The regression models also adjusted for APOE-ε4 carrier status (carrier versus non-carrier). In the overall sample, we tested for the 3-way interaction of sex*biomarker change*group (MCI vs. Preclinical AD). For the dichotomous variables of sex and group, females and Preclinical AD were the reference groups, respectively. We then examined interaction effects of sex*biomarker change in separate models for those with MCI and those in the preclinical stage.

We conducted a sensitivity analysis restricting the MCI group to those who are AD biomarker positive (CSF pTau_181_/Aβ_42_ ratio, amyloid PET or Tau PET biomarkers, *N* = 293). This approach increases the likelihood that the included MCI participants are on the AD trajectory, given the heterogeneity of MCI, and better aligns them with the biomarker-positive preclinical AD group while representing a more advanced, mildly impaired stage. Missing data were not replaced. Only individuals with complete data were included in the sample. In secondary analyses, we examined whether the results were specific to the memory domain by repeating analysis with the outcomes of Trail Making Test Part B (Trails B) scores, representing executive function performance, and the Clinical Dementia Rating Sum of Boxes (CDR-SB), a global measure of cognitive and functional impairment. The Trails B score is the time in seconds needed to complete the test with higher scores reflecting poorer performance (range: 0–300) and higher scores on the CDR-SB reflect greater cognitive and functional impairment (range: 0–18). Trails B and CDR-SB change scores were calculated by creating lagged residuals with a lag of one time point. Quantile regression was used with pTau_181_/Aβ_42_ = 0.5 to model median differences due to non-normal distributions in the dependent variables of Trails B and CDR-SB scores.

## Results

Table 1 summarizes sex differences in sample characteristics at baseline. Males were significantly more educated and had a significantly higher proportion of MCI cases than females. As expected, males had a significantly lower memory composite z-score than males at baseline. The also had a significantly lower memory composite change z-score than females indicative of greater memory decline overall. The average follow-up duration for both males and females was about 4 years after baseline.

**Table 1:** Sample characteristics at baseline by sex

	Females (*N* = 182)	Males (*N* = 219)	*p*-value
Age, M (SD)	73.6 (6.0)	74.9 (7.0)	0.20
Years of education, M (SD)	15.3 (2.6)	16.6 (2.7)	**0.002**
Race, % non-Hispanic white, N (%)	178 (97.7%)	212 (96.7%)	0.84
APOE-ε4 carrier, N (%)	107 (59.0%)	121(55.2%)	0.67
Diagnostic Group			**0.03**
Preclinical AD, N (%)	78 (43.0%)	73 (33.3%)	
MCI, N (%)	104 (57.0%)	146 (66.7%)	
Follow-up period (yrs), M (SD)	4.0 (2.4)	4.4 (2.8)	0.17
Progressed to dementia, N (%)	61 (33.3%)	81 (37.1%)	0.26
Baseline CSF pTau_181_/Aβ_42_ ratio, M (SD)	0.045 (0.031)	0.042 (0.024)	0.32
Baseline memory composite z-score, M (SD)	0.02 (0.89)	−0.74 (0.78)	**0.004**
CSF pTau_181_/Aβ_42_ change score, M (SD)^a^	0.055 (0.033)	0.053 (0.024)	0.42
Verbal memory composite change score, M (SD)^a^	0.116 (0.962)	−0.123 (0.887)	**0.003**

AD = Alzheimer’s disease, MCI = mild cognitive impairment, pTau = hyperphosphorylated tau, Aβ = amyloid-beta, APOE-ε4 = apolipoprotein E ε4 allele

Bold text indicates statistical significance at *p* <.05

^a^Higher absolute value indicates greater change, while negative values indicate decline and positive values indicate improvement

We examined unadjusted sex differences in mean baseline and change scores of CSF pTau_181_/Aβ_42_ levels and the memory composite z-score by diagnostic group. As expected, the mean memory composite z-score was significantly higher in females versus males in both the Preclinical AD and MCI groups. The mean memory composite change z-score was positive for both males and females in the Preclinical AD group suggestive of increasing performance over time due to practice effects; however, this mean was significantly higher in females versus males suggesting greater practice gains in memory performance in females versus males with Preclinical AD, which aligns with prior literature [30]. The mean memory composite change z-scores were negative and statistically similar for males and females in the MCI group suggesting memory decline for both sexes despite repeated assessment. The baseline CSF pTau_181_/Aβ_42_ levels did not significantly differ by sex within either diagnostic group; however, in the MCI group, a significantly higher CSF pTau_181_/Aβ_42_ change score in MCI females versus MCI males indicates a greater increase in CSF pTau_181_/Aβ_42_ levels females versus males in the MCI stage (Table 2).

**Table 2:** Average baseline and change scores for the AD biomarker and the memory composite z-score by diagnostic group and sex

	Preclinical AD			MCI		
	Females	Males	*p*-value	Females	Males	*p*-value
Baseline CSF pTau_181_/Aβ_42_ ratio, M (SD)	0.041 (0.018)	0.047 (0.022)	0.88	0.047 (0.032)	0.042 (0.026)	0.15
Baseline memory composite z-score, M (SD)	1.017 (0.903)	0.613 (0.843)	**< 0.001**	0.071 (0.985)	−0.246 (0.717)	**0.002**
CSF pTau_181_/Aβ_42_ change score, M (SD)^a^	0.042 (0.017)	0.047 (0.020)	0.10	0.066 (0.044)	0.056 (0.023)	**0.04**
Memory composite change z-score, M (SD)^a^	1.166 (0.730)	0.774 (0.854)	**0.003**	−0.328 (0.568)	−0.444 (0.587)	**0.12**

Two-sample *t*-tests were used to test sex differences in outcomes within diagnostic group. Abbreviations: AD = Alzheimer’s disease, MCI = mild cognitive impairment, pTau = hyperphosphorylated tau, Aβ = amyloid-beta, APOE-ε4 = apolipoprotein E ε4 allele

^a^Higher absolute value indicates greater change, while negative values indicate decline and positive values indicate improvement. Bold text indicates statistical significance

In the full sample, the sex* biomarker change*diagnostic group (Preclinical vs. MCI) interaction was significant (*b* = −16.63, 95%CI = −26.60 to −6.33, *p* =.002). Table 3 summarizes the full model. Evidence of the significant 3-way interaction indicates that the relationship between change in the pTau_181_/Abeta_42_ ratio and change in the verbal memory composite score significantly differed by sex and group status. The greater influence of change in the pTau_181_/Aβ_42_ ratio on change in verbal memory composite score was evident in the MCI group only, controlling for covariate effects.

**Table 3:** Results of model testing the diagnostic group * sex * pTau_181_/Aβ_42_ change interaction on the memory composite change z-score

Predictors	Memory Composite Change Z-Score			
	*b*	95% CI	*p*-value	Effect size
Intercept	0.48	0.21–0.74	**< 0.001**	−0.28 (−0.40, −0.17
pTau_181_/Aβ_42_ change score	−12.25	−16.27 – −8.22	**< 0.001**	−0.40 (−0.54, −0.27
Sex [female]	−0.33	−0.65 – −0.01	**< 0.05**	0.12, (−0.06, 0.30
Diagnostic group [Preclinical AD]	0.22	−0.18–0.62	0.280	0.52 (0.32, 0.73
Age (centered)	−0.01	−0.02–0.00	0.188	−0.05 (−0.12, 0.02)
Years of education (centered)	0.01	−0.02–0.03	0.690	0.01 (−0.06, 0.09)
APOE-ε4 carrier status [non ε4 carrier]	−0.12	−0.25–0.03	0.107	−0.06 (−0.13, 0.01)
pTau_181_/Aβ_42_ change score * Sex	8.27	3.45–13.10	**0.001**	0.27 (0.11, 0.43)
pTau_181_/Aβ_42_ change score * Diagnostic group	5.10	−1.68–11.88	0.140	0.17 (−0.06, 0.39)
Sex * Diagnostic group	1.04	0.47–1.61	**< 0.001**	0.15 (−0.16, 0.46)
pTau_181_/Aβ_42_ change score * Sex * Diagnostic group	−16.63	−26.92 – −6.33	**0.002**	−0.55 (−0.89, −0.21)
**Random Effects**				
σ^2^	0.57			
τ_00 ID_	0.12			
ICC	0.17			
N	401			
R^2^	0.348			

Reference groups of dichotomous variables are indicated in brackets

CI = confidence interval, pTau = hyperphosphorylated tau, Aβ = amyloid-beta, APOE-ε4 = apolipoprotein E ε4 allele. ICC = intraclass correlation coefficient. *b* = unstandardized regression weights

Effect size is represented by standardized regression weights and their corresponding 95% confidence (0.00–0.19.00.19 is a small effect; 0.20–0.39 is a moderate effect; ≥0.40 is a large effect). Bold text indicates statistical significance at *p* <.05

**Fig. 1:**
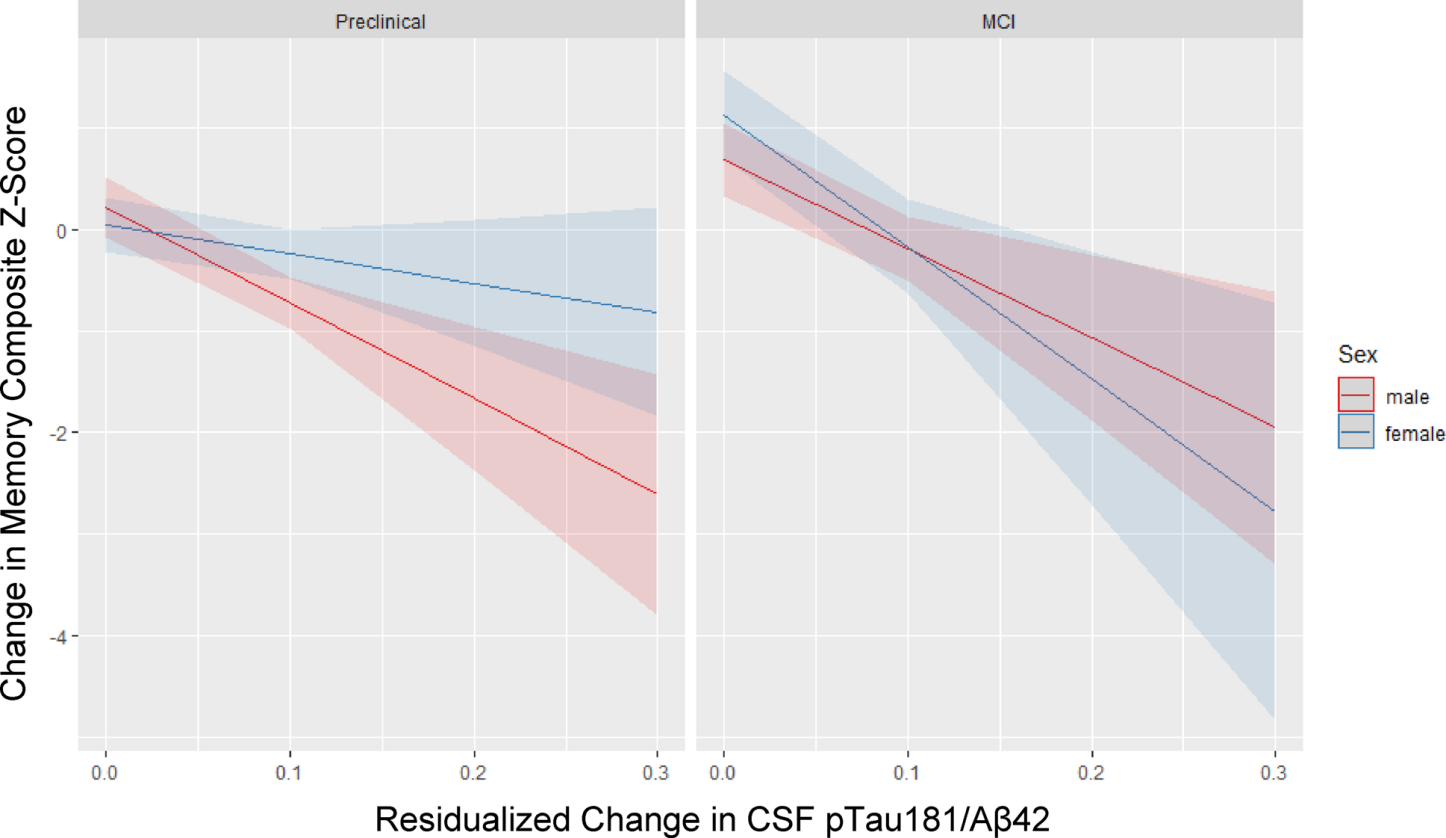
Sex differences in the relationship between change in the CSF pTau181/Aβ42 ratio and change in the memory composite z-score by baseline diagnostic group. Males are in red and females are in blue. Those classified as Preclinical AD at baseline are pictured in the left panel and those classified as MCI at baseline are pictured in the right panel. Higher memory composite z-scores reflect better performance. Higher pTau181/Aβ42 levels reflect greater pathology. The average follow-up duration for both males and females is approximately 4 years

Results from regression models separated by preclinical AD and MCI status are in Table 4 and visually depicted in Fig. 1. In the preclinical group, change in pTau_181_/Abeta_42_ ratio was not a significant predictor of change in the verbal memory composite score (*b* = 2.55, 95%CI = −12.61 to 18.01, *p* =.75). The wide confidence interval indicates a lack of precision in the measured effect between these two change processes. Although the pattern of results was as hypothesized (i.e., females showing less memory decline than males as pTau_181_/Aβ_42_ increased), the interaction was not significant (*b* = −9.73, 95%CI = −19.78 to 1.60, *p* =.09) but the main effect of sex (*b* = 0.75, 95%CI = 0.19 to 1.31, *p* =.009) was significant. In contrast, the MCI model demonstrated a different pattern of results. There was a significant negative relationship between change in the pTau_181_/Aβ_42_ ratio and change in the verbal memory composite score (*b* = −20.80, 95% CI −28.13 to −12.79, *p* <.001). There was also a significant main effect of sex indicating a significantly steeper decline in the verbal memory composite score among females (*b* = −0.35, 95%CI = −0.64 to −0.06, *p* =.019). A significant sex * biomarker change interaction (*b* = 8.36, 95% CI = 3.99 to 12.72, *p* <.001) indicated that females experienced a stronger influence of change in the pTau_181_/Abeta_42_ ratio than males on their decline of the verbal memory composite score.

**Table 4:** Comparison of diagnostic group models for change in memory composite z-score

	Preclinical Group - Memory Composite Change Z-Score				MCI Group – Memory Composite Change Z-Score			
	*b*	95% CI	*p*-value	Effect size	*b*	95% CI	*p*-value	Effect size
Intercept	0.63	0.23–1.04	**0.002**	−0.06 (−0.19, 0.07)	0.52	−0.06–0.19	**< 0.001**	−0.20 (−0.38, −0.02)
pTau_181_/Aβ_42_ change score	−7.23	−13.64−0.81	**0.027**	−0.52 (−0.67, −0.36	−12.20	−32.78 – −15.18	**< 0.001**	−0.17 (−0.32, −0.02)
Sex [female]	0.72	0.16–1.27	**0.012**	0.15 (−0.05, 0.35)	−0.36	−0.17 – −0.03	**0.015**	0.33 (0.07, 0.59)
Age (centered)	−0.01	−0.03–0.02	0.699	−0.08 (−0.18, 0.02)	−0.01	−0.01–0.00	0.130	−0.03 (−0.16, 0.11)
Years of education (centered)	0.03	−0.02–0.08	0.261	−0.02 (−0.12, 0.08)	−0.01	−0.02–0.00	0.668	0.08 (−0.06, 0.21)
APOE-ε4 carrier status [non ε4 carrier]	−0.02	−0.29–0.25	0.872	−0.10 (−0.19, 0.00)	−0.16	−0.03–0.11	0.057	−0.01 (−0.14, 0.12)
pTau_181_/Aβ_42_ change score * Sex	−8.05	−18.76–2.65	0.140	0.36 (0.17, 0.54)	8.45	4.94–15.40	**< 0.001**	−0.19 (−0.43, 0.06)
**Random effects**								
σ^2^	0.81				0.43			
τ_00 ID_	0.12				0.14			
ICC	0.12				0.24			
N	138				263			
R^2^	0.219				0.325			

Reference groups of dichotomous variables are indicated in brackets

MCI = mild cognitive impairment, CI = confidence interval, pTau = hyperphosphorylated tau, Aβ = amyloid-beta, APOE-ε4 = apolipoprotein E ε4 allele. ICC = intraclass correlation coefficient. *b* = unstandardized regression weights

Effect size is represented by standardized regression weights and their corresponding 95% confidence intervals (0.00–0.19.00.19 is a small effect; 0.20–0.39 is a moderate effect; ≥0.40 is a large effect). Bold text indicates statistical significance at *p* <.05

In addition to stratifying by diagnostic group to probe the three-way diagnostic group * sex * pTau181/Aβ24 change score interaction, we also present results stratified by sex to provide complementary insights and a more complete characterization of these relationships (see Supplemental Table 1). The two-way diagnostic group * pTau181/Aβ24 change score interaction was only significant in females (*b* = −11.61, 95% CI = −19.46 to −3.77, *p* =.004), whereby increases in the pTau181/Aβ24 ratio related to greater decreases in the memory composite z-score in females with MCI versus females with Preclinical AD. In males, there was a significant relationship between increases in the pTau181/Aβ24 ratio and decreases in the memory composite z-score (b = −12.20, 95% CI = −16.23 to −8.18, *p* <.001) that did not statistically differ by diagnostic group.

In sensitivity analyses limiting the MCI sample to those who are AD biomarker positive (*N* = 293), the results did not change (results not shown).

In secondary analyses examining change in Trails B and CDR-SB scores as the outcomes, the sex*biomarker change*diagnostic group interactions were no longer significant, nor were the two-way interactions of sex*biomarker change or sex*diagnostic group or the main effect of sex (*p*s > 0.05). In the Trails B model, there was a significant biomarker change*diagnostic group interaction (*b* = 585.71, 95% CI 30.23 to1141.19, *p* =.039) that indicated that increases in pTau_181_/Aβ_42_ levels related to greater increases in Trails B scores (decreasing executive function performance) in the MCI group compared to the Preclinical AD group (Supplemental Tables 2 and Supplemental Fig. 1). In the CDR-SB model, there was only a main effect of biomarker change (*b* = 22.67, 95% CI = 9.19 to 36.15, *p* =.002), whereby increases in pTau_181_/Aβ_42_ related to increases in CDR-SB scores (increasing cognitive impairment) (Supplemental Tables 3 and Supplemental Fig. 2).

## Discussion

This is the first longitudinal study to demonstrate sex differences in how changes in AD biomarkers related to changes in memory and that the direction of these differences depends on the preclinical versus prodromal stages of AD. In line with hypotheses, we found that males in the preclinical AD group showed steeper memory decline as the pTau_181_/Aβ_42_ ratio levels advanced compared to females in the preclinical AD group, but this difference was not statistically significant perhaps due, in-part, to the smaller sample size and consequent lower statistical power and greater variability in the preclinical AD group. The pattern of results in the MCI group also align with hypothesis in that females showed significantly steeper memory decline as the pTau_181_/Aβ_42_ ratio levels advanced compared to males. This finding suggests that females with MCI experience a steeper decrease in memory function per unit of increasing pTau_181_/Aβ_42_ ratio than males and aligns with past studies reporting greater cognitive decline in females compared to males with MCI [13–15]. Overall, the pattern of results suggests that females are better able than males to maintain normal memory performance despite early AD pathogenesis; however, there seems to be a tipping point, whereby, when AD pathogenesis reaches more advanced stages, it has a more detrimental impact on memory decline compared to males. These sex differences did not extend to the Trails B and the CDR-SB suggesting that they are specific to the memory domain and perhaps more specifically to verbal memory.

The pattern of a female memory advantage in preclinical stages followed by faster decline in later stages has been suggested by prior cross-sectional studies showing a memory advantage in females among those with mild AD biomarker burden (i.e., Aβ, tau, hippocampal volume, brain glucose metabolism) that disappeared among individuals with moderate-to-severe pathological burden, suggestive of a steeper decline in females [8, 9, 11, 31, 32]. Studies by Caldwell et al. specifically examined the influence of sex at the preclinical AD stage. They found significantly worse verbal memory performance in cognitively normal males who were PET-derived Aβ biomarker positive versus biomarker negative, whereas this difference was absent in females [7, 8], suggesting that verbal memory function in females is more resilient to the adverse effects of early-stage AD pathogenesis, resulting in less decline at this stage.

Longitudinal studies have supported the steeper decline in females with MCI. Previously in ADNI, Lin et al. found two fold faster decline in females with MCI compared to males with MCI on the global cognitive tests of the Alzheimer’s Disease Assessment Scale–Cognitive Subscale (ADAS-COG) and the Clinical Dementia Rating-Sum of Boxes (CDR-SOB) [14]. In 688 ADNI participants, Holland et al. compared sex/gender differences in cognitive and brain volume decline among cognitively normal, MCI and AD dementia groups [13]. They found that, despite females showing steeper declines in hippocampus, entorhinal cortex and amygdala volumes in the cognitively normal group, steeper cognitive decline (ADAS-Cog and CDR-SOB performance) in females was only found in the MCI and AD dementia groups [13]. This pattern of results again suggests memory performance that is more resilient to early AD pathogenic changes in females compared to males, whereas the opposite is true in later stages. Our current study is a much-needed next step in this literature by demonstrating longitudinally that how memory function is affected by advancing AD pathology depends on sex and that these sex differences are not uniform across the AD trajectory.

Our results are also consistent with recent findings from Memel et al. (2025) in a large autosomal dominant frontotemporal dementia (FTD) cohort from the ALLFTD Consortium. In that study, females showed cognitive resilience on a global composite (memory, language, executive function) in asymptomatic stages followed by more rapid decline than males in symptomatic stages, despite similar levels of neurodegeneration markers early on [33]. This parallel pattern across two distinct neurodegenerative diseases suggests that such stage-dependent sex differences in clinical trajectories may not be AD-specific, but rather may reflect broader, sex-related mechanisms of resilience and vulnerability in the face of advancing neurodegeneration.

Sex differences in the clinical presentation of AD pathology have critical research and clinical implications. One such implication is the vital role of AD biomarkers in early detection and disease tracking that is swiftly expanding as technology advances [34]. Biomarker research and clinical use has, for the most part, operated under the assumption that biomarkers and their cut-points reflect similar disease risk, stage and progression in males and females. Diagnostic thresholds for biomarkers and our theoretical models of the temporal progression of biomarkers will likely be improved if sex-specific patterns are accounted for. Understanding sex differences in the link between AD biomarkers and clinical trajectories and incorporating sex-specific considerations into guidelines for biomarker-based tools can lead to earlier and more accurate diagnoses, improved disease monitoring and better-targeted interventions for both males and females. Other critical implications revolve around evidence from the current and past findings suggesting that females are at a more advanced disease stage than males, pathologically, when diagnosed with MCI [9–11, 35]. Supportively, in a hospital-based clinical sample of 349 older adults diagnosed with MCI, Karstens et al. found that females showed significantly worse performance in non-memory domains (language and executive functioning/information processing speed) compared to males despite their comparable performance on MCI criteria, global cognitive screening tasks and memory domain performance [36]. This results in limited opportunities for early diagnosis and intervention in females when our currently-available pharmaceutical and non-pharmaceutical interventions have the greatest potential of altering the disease course. Lastly, the differing pattern of sex differences by disease stage indicates the importance of accounting for disease stage when conducting research examining sex differences in AD. This is particularly true for cross-sectional studies, in which sex difference findings may be masked when examined across preclinical, MCI and AD dementia stages.

We can only speculate as to the reasons behind the sex differences in the clinical presentation of AD pathology. They are likely multi-factorial but one critical factor is the life-long female advantage in verbal memory, particularly given the specificity of results to the verbal memory outcome. This female advantage has vital clinical implications in the context of AD given that verbal memory tests are central to MCI and AD dementia diagnostic protocols. Because normative data for these tests often do not account for baseline sex differences, detection of early verbal memory decline in females is more challenging. Indeed, studies that compared MCI diagnosis rates resulting from MCI criteria involving sex-adjusted versus non-sex-adjusted verbal memory normative data found that the sex-adjusted criteria resulted in more MCI cases in females and less MCI cases in males compared to the non-sex-adjusted criteria [37–39]. This female advantage in verbal memory also means that they have further to fall on the AD trajectory.

There may be biological mechanisms that support female’s cognitive resilience to early AD pathology followed by their steeper decline thereafter. For instance, greater brain glucose metabolism in females compared to males that is sustained at early, but not later, AD stages could potentially provide greater resilience against early AD-related brain changes [40–45]. Additionally, in a post-mortem study examining translocator protein (TSPO) levels in brain tissue as a marker of neuroinflammation, Acosta-Martinez et al. found that, among females but not males, there were significant, region-dependent elevations of TSPO density in AD dementia cases compared to MCI and cognitively normal cases and a significant, positive correlation between TSPO density and tau burden [46]. A stronger neuroinflammatory response in females with AD relative to males was also reported recently by Biechele et al. [47]. These findings raise the possibility that increases in neuroinflammation later in the AD trajectory may contribute to female’s steeper cognitive decline.

Our study has limitations. ADNI is a convenience sample of mostly white and well-educated volunteers, which limits generalizability of results. It is imperative to examine this research question in more diverse samples that better reflect the U.S. population and understand how social determinants of health influence sex/gender differences in AD. It would be informative to repeat our analyses with a visual memory test to see how results compare with a memory task that does not show a sex bias; however, this data is unavailable in ADNI. Lastly, our sample size and, in turn, statistical power was limited once stratifying by diagnostic group, particularly in the preclinical group where the sex difference pattern was as hypothesized but not statistically significant.

## Conclusions

In conclusion, we found sex differences in how changes in biomarkers of AD pathology relate to verbal memory decline and that these differences vary by disease stage. Although the moderating role of sex was not significant in the preclinical AD group, the pattern of results suggests that females show less memory decline than males in response to advancing AD pathology. However, in the MCI stage, the sex difference was in the opposite direction, whereby females showed significantly steeper memory decline than males in response to advancing AD pathology. While this change in sex disparities in the AD pathology and symptomology link was suggested by prior cross-sectional studies, this longitudinal study is a more definitive test of this hypothesis. Overall, the change in sex differences highlights a possible “tipping point” in females, where resilience transitions to vulnerability as the disease progresses. These findings have critical clinical and research implications. They emphasize the need for sex-specific approaches to AD biomarker-based diagnostics and interventions to improve outcomes for both sexes, especially in early AD detection in the higher risk sex, females.

## Supplementary Information

Supplementary Material 1

Supplementary Material 2

## Data Availability

The dataset supporting the conclusions of this article is available in the ADNI data repository, [https://adni.loni.usc.edu/data-samples/adni-data/]. All ADNI data are shared through the LONI Image and Data Archive (IDA), a secure research data repository. Interested scientists may obtain access to ADNI imaging, clinical, genomic, and biomarker data for the purposes of scientific investigation, teaching, or planning clinical research studies. Access is contingent on adherence to the ADNI Data Use Agreement and the publication policies outlined in the documents available at https://adni.loni.usc.edu/data-samples/adni-data/.
