# Supplementary material for: Sex differences in the relationship of biomarker change to memory decline in early Alzheimer’s disease: an observational cohort study
Source: Biol Sex Differ. 2026 Jan 16;17:38. doi: 10.1186/s13293-025-00820-6 (PMC12930658; doi:10.1186/s13293-025-00820-6)
Supplement: Supplementary file 1 — Supplementary Material 1 [file 13293_2025_820_MOESM1_ESM.docx]

Supplemental Table 1. Comparison of models examining change in the pTau_181_/Aβ_42_ ratio in relation to change in memory composite z-score by sex.

|  | Females - Memory Composite Change Z-Score | | | Males - Memory Composite Change Z-Score | | |
| --- | --- | --- | --- | --- | --- | --- |
| Predictors | *b* | 95% CI | p-value | *b* | 95% CI | p-value |
| (Intercept) | 0.22 | -0.02 – 0.47 | 0.071 | 0.40 | 0.12 – 0.68 | **0.006** |
| pTau_181_/Aβ_42_ change score | -3.85 | -6.51 – -1.19 | **0.005** | -12.20 | -16.23 – -8.18 | **<0.001** |
| Diagnostic group [Preclinical AD] | 1.28 | 0.86 – 1.69 | **<0.001** | 0.21 | -0.19 – 0.61 | 0.304 |
| Age (centered) | -0.02 | -0.04 – -0.00 | **0.030** | -0.00 | -0.02 – 0.01 | 0.759 |
| Years of education (centered) | -0.02 | -0.05 – 0.02 | 0.353 | 0.02 | -0.01 – 0.05 | 0.268 |
| APOE-ε4 carrier status [non ε4 carrier] | -0.29 | -0.51 – -0.07 | **0.011** | -0.02 | -0.21 – 0.17 | 0.820 |
| pTau_181_/Aβ_42_ change score × Diagnostic group | -11.61 | -19.46 – -3.77 | **0.004** | 5.25 | -1.51 – 12.00 | 0.127 |
| **Random Effects** | | | | | | |
| σ^2^ | 0.60 | | | 0.55 | | |
| τ_00_ | 0.09 | | | 0.13 | | |
| ICC | 0.13 | | | 0.19 | | |
| N | 184 | | | 217 | | |
| Observations | 327 | | | 418 | | |
| Marginal R^2^ / Conditional R^2^ | 0.260 / 0.356 | | | 0.165 / 0.328 | | |

Table Legend. Reference groups of dichotomous variables are indicated in brackets. Abbreviations: CI=confidence interval, pTau=hyperphosphorylated tau, Aβ=amyloid-beta, APOE-ε4=apolipoprotein E ε4 allele. ICC=intraclass correlation coefficient. *b*=unstandardized regression weights. Bold text indicates statistical significance at p<.05.

Supplemental Table 2. Results of model testing the diagnostic group * sex * pTau_181_/Aβ_42_ change interaction on the Trails B change score.

|  | Trails B Change Score | | | |
| --- | --- | --- | --- | --- |
| *Predictors* | *b* | *95% CI* | *p-value* | *Effect Size* |
| Intercept | 121.38 | 99.56 – 143.20 | **<0.001** | 0.10 (-0.01, 0.20) |
| pTau_181_/Aβ_42_ change score | 335.94 | 8.43 – 663.46 | **0.044** | 0.24 (0.12, 0.36) |
| Sex [female] | -0.89 | -26.87 – 25.08 | 0.946 | -0.15 (-0.32, 0.01) |
| Diagnostic group [Preclinical AD] | -51.78 | -84.50 – -19.06 | **0.002** | -0.14 (-0.25, -0.03) |
| Age (centered) | 0.54 | -0.32 – 1.41 | 0.218 | 0.05 (-0.03, 0.13) |
| Years of education (centered) | 2.24 | 0.21 – 4.28 | **0.031** | 0.09 (0.01, 0.17) |
| APOE-ε4 carrier status [non ε4 carrier] | -10.69 | -22.33 – 0.95 | 0.072 | -0.07 (-0.15, 0.01) |
| pTau_181_/Aβ_42_ change score * Sex | -207.08 | -598.64 – 184.47 | 0.299 | -0.10 (-0.26, 0.07) |
| pTau_181_/Aβ_42_ change score * Diagnostic group | 585.71 | 30.23 – 1141.19 | **0.039** | 0.12 (0.01. 0.24) |
| Sex * Diagnostic group | 5.03 | -40.98 – 51.04 | 0.830 | 0.03 (-0.14, 0.19) |
| pTau_181_/Aβ_42_ change score * Sex * Diagnostic group | -22.48 | -856.59 – 811.62 | 0.958 | -0.005 (-0.18, 0.17) |
| **Random Effects** | | | | |
| σ^2^ | 3607.95 | | | |
| τ_00_ _RID_ | 907.27 | | | |
| ICC | 0.20 | | | |
| N _RID_ | 402 | | | |
| Observations | 742 | | | |
| Marginal R^2^ / Conditional R^2^ | 0.083 / 0.268 | | | |

Table Legend. Reference groups of dichotomous variables are indicated in brackets. Abbreviations: Trails B = Trail Making Test, Part B, CI=confidence interval, pTau=hyperphosphorylated tau, Aβ=amyloid-beta, APOE-ε4=apolipoprotein E ε4 allele. ICC=intraclass correlation coefficient. *b*=unstandardized regression weights. Effect size is represented by standardized regression weights and their corresponding 95% confidence intervals (0.00-0.19 is a small effect; 0.20-0.39 is a moderate effect; ≥0.40 is a large effect). Bold text indicates statistical significance at p<.05.

Supplemental Table 3. Results of model testing the diagnostic group * sex * pTau_181_/Aβ_42_ change interaction on the CDR-SB change score.

|  | CDR-SB Change Score | | | |
| --- | --- | --- | --- | --- |
| *Predictors* | *b* | *95% CI* | *p-value* | *Effect Size* |
| Intercept | 0.48 | -0.22 – 1.19 | 0.182 | -0.16 (-0.24, -0.07) |
| pTau_181_/Aβ_42_ change score | 22.67 | 9.19 – 36.15 | **0.002** | 0.26 (0.12, 0.40) |
| Sex [female] | 0.30 | -0.44 – 1.03 | 0.429 | -0.08 (-0.19, 0.04) |
| Diagnostic group [Preclinical AD] | -0.96 | -2.04 – 0.12 | 0.087 | -0.14 (-0.29, 0.00) |
| Age (centered) | -0.01 | -0.03 – 0.01 | 0.332 | -0.02 (-0.07, 0.03) |
| Years of education (centered) | 0.02 | -0.03 – 0.06 | 0.466 | 0.02 (-0.03, 0.08) |
| APOE-ε4 carrier status [non ε4 carrier] | -0.05 | -0.30 – 0.19 | 0.670 | -0.02 (-0.06, 0.02) |
| pTau_181_/Aβ_42_ change score * Sex | -9.28 | -23.65 – 5.10 | 0.211 | 0.05 (-0.12, 0.21) |
| pTau_181_/Aβ_42_ change score * Diagnostic group | 9.47 | -13.98 – 32.92 | 0.432 | -0.02 (-0.21, 0.16) |
| Sex * Diagnostic group | -0.32 | -1.46 – 0.82 | 0.587 | 0.02 (-0.15, 0.20) |
| pTau_181_/Aβ_42_ change score * Sex * Diagnostic group | 3.89 | -22.15 – 29.94 | 0.770 | 0.03 (-0.18, 0.24) |
| **Random Effects** | | | | |
| σ^2^ | 0.02194 | | | |
| N _RID_ | 404 | | | |
| Observations | 758 | | | |

Table Legend. Reference groups of dichotomous variables are indicated in brackets. Abbreviations: CDR-SB = Clinical Dementia Rating – Sum of Boxes, CI=confidence interval, pTau=hyperphosphorylated tau, Aβ=amyloid-beta, APOE-ε4=apolipoprotein E ε4 allele. ICC=intraclass correlation coefficient. *b*=unstandardized regression weights. Effect size is represented by standardized regression weights and their corresponding 95% confidence intervals (0.00-0.19 is a small effect; 0.20-0.39 is a moderate effect; ≥0.40 is a large effect). Bold text indicates statistical significance at p<.05.
