# Supplementary material for: Sex differences in the relationship of biomarker change to memory decline in early Alzheimer’s disease: an observational cohort study
Source: Biol Sex Differ. 2026 Jan 16;17:38. doi: 10.1186/s13293-025-00820-6 (PMC12930658; doi:10.1186/s13293-025-00820-6)
Supplement: Supplementary file 2 — Supplementary Material 2 [file 13293_2025_820_MOESM2_ESM.docx]

Supplement Figure 1.


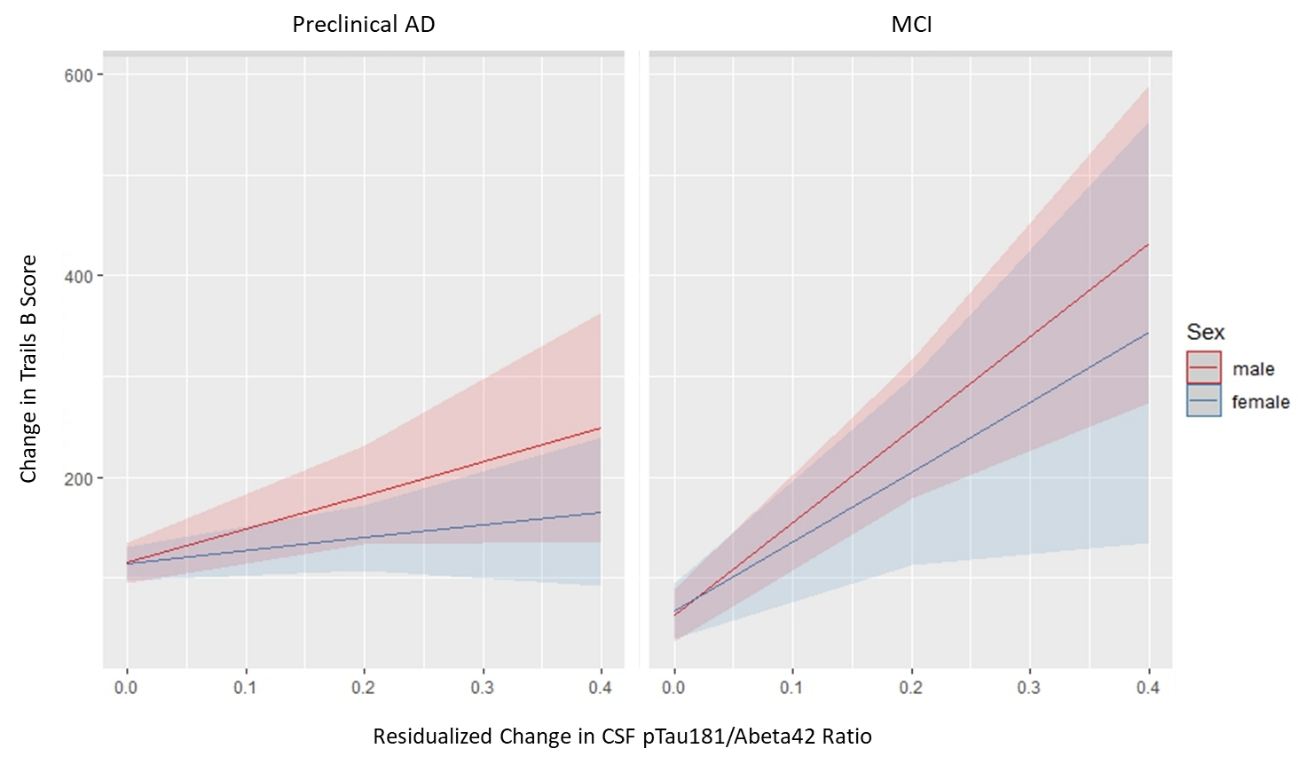
 Supplemental Figure 1. Relationship between change in pTau181/Aβ42 ratio and change in the Trail Making Test Part B (Trails B) score by sex and baseline diagnostic group. Males are in red and females are in blue. Those classified as Preclinical AD at baseline are pictured in the left panel and those classified as MCI at baseline are pictured in the right panel. Higher Trails B scores reflect worse performance. Higher pTau181/Aβ42 levels reflect greater pathology. The average follow-up duration for both males and females is approximately 4 years.

Supplement Figure 2.


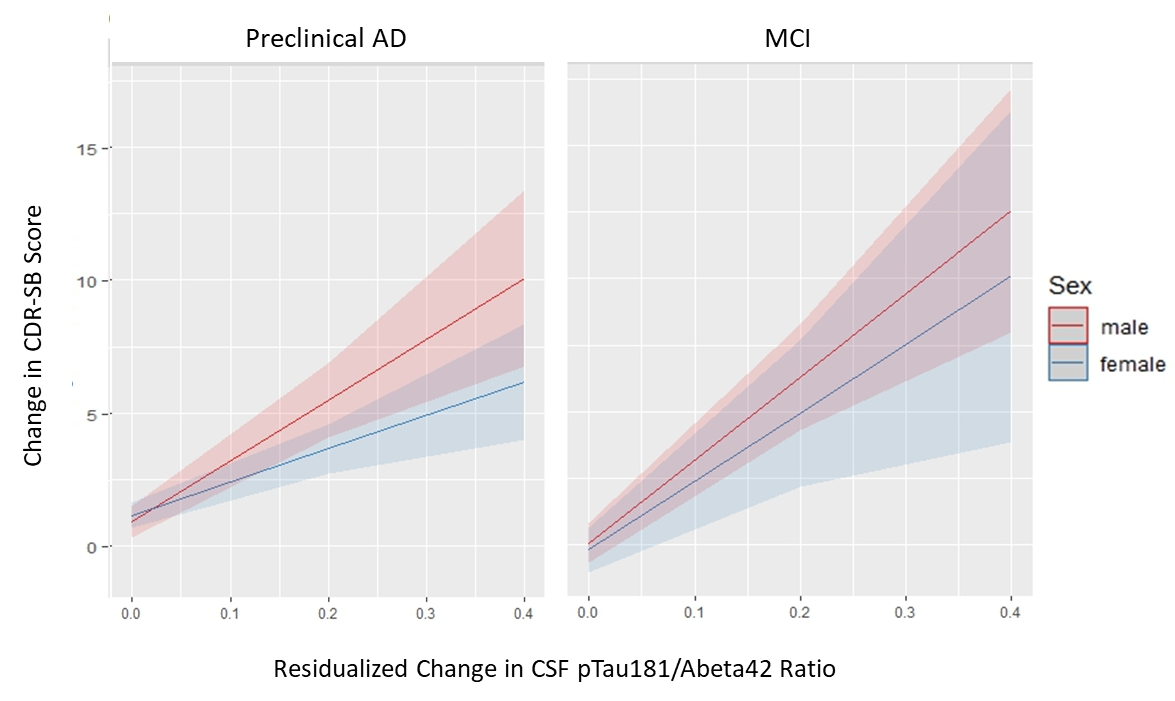


Supplemental Figure 2. Relationship between change in pTau181/Aβ42 ratio and change in the Clinical Dementia Rating – Sum of Boxes (CDR-SB) by sex and baseline diagnostic group. Males are in red and females are in blue. Those classified as Preclinical AD at baseline are pictured in the left panel and those classified as MCI at baseline are pictured in the right panel. Higher CDR-SB scores reflect greater impairment. Higher pTau181/Aβ42 levels reflect greater pathology. The average follow-up duration for both males and females is approximately 4 years.
